# INTS7–ABCD3 Interaction Stimulates the Proliferation and Osteoblastic Differentiation of Mouse Bone Marrow Mesenchymal Stem Cells by Suppressing Oxidative Stress

**DOI:** 10.3389/fphys.2021.758607

**Published:** 2021-11-22

**Authors:** Yubo Liu, Xiao Yu, Anquan Huang, Xiangxin Zhang, Yijun Wang, Wei Geng, Renjie Xu, Suoyuan Li, Hui He, Bo Zheng, Guangxiang Chen, Yaozeng Xu

**Affiliations:** ^1^Department of Orthopaedics, The First Affiliated Hospital of Soochow University, Suzhou, China; ^2^Department of Orthopaedics, The Affiliated Suzhou Hospital of Nanjing Medical University, Suzhou, China; ^3^State Key Laboratory of Reproductive Medicine, Center for Reproduction and Genetics, Suzhou Municipal Hospital, The Affiliated Suzhou Hospital of Nanjing Medical University, Gusu School, Nanjing Medical University, Suzhou, China; ^4^State Key Laboratory of Reproductive Medicine, Department of Histology and Embryology, Nanjing Medical University, Nanjing, China

**Keywords:** bone marrow mesenchymal stem cells (BM-MSCs), INTS7, ABCD3, proliferation, osteoblastic differentiation, adipogenic differentiation, oxidative stress

## Abstract

Increased adipocyte and decreased osteoblast differentiation, combined with the ectopic proliferation of bone marrow mesenchymal stem cells (BM-MSCs), represent the primary causes of osteoporosis. The dysregulation of numerous intracellular bioactive factors is responsible for the aberrant differentiation and growth of BM-MSCs. In this study, we focused on a new stimulative factor, integrator complex subunit 7 (INTS7), and its cooperative protein ATP-binding cassette subfamily D member 3 (ABCD3)/high-density lipoprotein-binding protein (HDLBP) in mouse BM-MSCs. We aimed to uncover the effects of the INTS7–ABCD3/HDLBP interaction on BM-MSC biological behaviors and the potential mechanism underlying these effects. Functional *in vitro* experiments showed that the suppression of the INTS7–ABCD3 interaction rather than HDLBP could impair BM-MSC proliferation and induce cell apoptosis. Moreover, Alizarin Red S and Oil Red O staining, respectively, revealed that INTS7 and ABCD3 knockdown but not HDLBP knockdown could decrease osteoblastic differentiation and accelerate the adipogenic differentiation of BM-MSCs. Mechanistically, reactive oxygen species (ROS) and histone γ-H2AX quantities significantly increased, whereas the levels of antioxidants declined due to INTS7 and ABCD3 inhibition in BM-MSCs. These findings indicated that the suppression of oxidative stress could be involved in the INTS7/ABCD3 co-regulatory mechanisms for BM-MSC proliferation and differentiation, identifying new potential candidates for osteoporosis therapy.

## Introduction

Osteoporosis is a systemic and progressive bone disease generally associated with aging that has become one of the most common and expensive diseases worldwide (Johnell and Kanis, 2006; Burge et al., 2007). Degraded bone microstructure, decreased bone mass, increased bone brittleness, and increased fracture risk are recognized as pervasive clinical characteristics of osteoporosis (Mulder et al., 2006; Pietschmann et al., 2009). Evidence suggests that mesenchymal stem cells (MSCs) play vital roles in initial bone formation, the maintenance of bone ossification, and fracture repair (Bielby et al., 2007). Bone marrow MSCs (BM-MSCs) are MSCs that reside in the bone marrow and are capable of differentiating into osteoblasts and adipocytes. During osteoporosis, BM-MSCs more frequently differentiate into adipocytes than osteoblasts, leading to a reduction in bone formation and an increase in the accumulation of marrow fat (Moerman et al., 2004; Li et al., 2015). Due to self-renewal capabilities and multiple differentiation potential, BM-MSCs have become effective candidates for cell-based osteoporosis therapy (Wang et al., 2012; Jiang et al., 2021).

Research has shown that the dysregulation of numerous intracellular bioactive factors (including transcription factors, signaling molecules, and microRNAs) contributes to the aberrant proliferation and differentiation of BM-MSCs (Hu et al., 2018). We examined two key transcription factors that regulate osteoblastic differentiation, runt-related transcription factor 2 (*Runx2*) and *Osterix*, which are both required for osteogenesis (Augello and De Bari, 2010). The study by Kobayashi et al. (2000) emphasized that *Runx2*-deficient cells failed to acquire osteoblastic phenotypes but presented with adipocytic phenotypes, associated with the increased expression of adipogenic markers (Kobayashi et al., 2000). *Osterix* can be activated by *Runx2*, and no osteoblastic differentiation of BM-MSCs or associated bone formation could be detected in *Osterix*-null mice (Nakashima et al., 2002; Yang et al., 2018). Additionally, peroxisome proliferation–activated receptor γ (PPARγ) and CCAAT/enhancer-binding protein α (C/EBPα) were both identified to promote adipocyte differentiation among BM-MSCs (Lane et al., 1996; Xu et al., 2016; Zhuang et al., 2016). β-catenin–dependent Wnt and transforming growth factor (TGF)-β superfamily signaling have both been characterized as essential pathways for BM-MSC differentiation and osteogenesis (Augello and De Bari, 2010). Additional bioactive factors that play dual roles in both osteoblast and adipocyte differentiation among BM-MSCs should be identified, and their underlying functions and mechanisms should be fully explored to better understand the balance between these two differentiation pathways.

In the present study, we explored the role played by the integrator complex subunit 7 (INTS7), a novel bioactive molecule, in mouse BM-MSC progression, for the first time. Integrator complex subunit 7 (INST7) has ever been identified as highly mutated and significantly overexpressed in diverse human cancers (Federico et al., 2017). Here, the effects of INTS7 on BM-MSCs proliferation and apoptosis were uncovered. Meanwhile, as a classical cell model, BM-MSCs possess self-renewal capabilities and multiple differentiation potential. Thus, whether the differentiation capacity of BM-MSCs was influenced by INTS7 regulation and the potential mechanisms were both explored here. A mass spectrometry analysis suggested the existence of an endogenous INTS7–ATP-binding cassette subfamily D member 3 (ABCD3)/high-density lipoprotein–binding protein (HDLBP) interaction in BM-MSCs. Functional *in vitro* experiments showed that the suppression of ABCD3 but not HDLBP could impair BM-MSC proliferation and induce cell apoptosis. Moreover, the stimulative efficacy of INTS7 and ABCD3 (but not HDLBP) for osteoblastic differentiation and their inhibitory impacts on adipocyte differentiation were further demonstrated in mouse BM-MSCs. Previous reports indicated that “increased reactive oxygen species (ROS) could inhibit MSCs proliferation and osteogenic differentiation, but enhance adipogenic differentiation” (Denu and Hematti, 2016), and our research further explored whether oxidative stress suppression was involved in the INTS7–ABCD3 co-regulatory axis for BM-MSC proliferation and differentiation.

## Materials and Methods

### Cell Culture and Transfection Reagents

The Oricell Strain C57BL/6 Mouse BM-MSCs (No: MUBMX-01001) were purchased from Cyagen Biosciences (Guangzhou, China) and cultured in Oricell C57BL/6 Mouse BM-MSCs Complete Medium (No: MUBMX-90011) supplemented with 440 ml basal medium, 50 ml qualified fetal bovine serum, 5 ml penicillin-streptomycin, and 5 ml glutamine. Cells were maintained in a water-saturated atmosphere at 37°C in 5% CO_2_. Cells were identified by the supplier according to the presence of cell surface markers and multipotency. Additionally, previous studies by other investigators have confirmed the stem cell identity of C57BL/6 Mouse BM-MSCs (Liu et al., 2013; Chen et al., 2020). An *Ints7* translation-blocking Vivo-morpholino (MO, Oligo Sequence: TTGACGCCATGACCCGGACAGTTAC) and a negative control MO (Oligo Sequence: CCTCTTACCTCAGTTACAATTTATA) were purchased from Gene Tools LLC (Philomath, OR, United States). The *Ints7* MO oligos bind to complementary RNA and block translation initiation in the cytosol by targeting the 5′ untranslated region (UTR) through the first 25 bases of the coding sequence. *Abcd3* short hairpin RNA (shRNA, Oligo Sequence: UUGAAAUCUUUGCUGCUGC), *Hdlbp* shRNA (Oligo Sequence: AUCCUUGUAGGUUGGAGGG), and a negative control shRNA (Oligo Sequence: ACGUGACACGUUCGGAGAA), were purchased from GenePharma (Shanghai, China) and transfected into BM-MSCs with Lipofectamine 2000 (Invitrogen, United States). *Abcd3*/*Hdlbp* shRNAs are plasmid vector-based shRNAs capable of specifically degrading target mRNAs *via* complementary binding sequences. Second to third passage BM-MSCs were used for experiments in this study.

### RNA Extraction and QRT-PCR Assays

The RNeasy Plus Micro Kit (Qiagen, Düsseldorf, Germany) was used to extract total RNA from BM-MSCs, according to the manufacturer’s instructions. RNA was reverse transcribed into cDNA using the PrimeScript Reverse Transcription Kit (Vazyme, Nanjing, China). SYBR green-based quantitative PCR was performed using an ABI 7500 machine (Applied Biosystems, Foster City, CA, United States). All results were normalized against 18S rRNA expression. The primers used in this study are summarized in Supplementary Table 1.

### Western Blot Assays and Antibodies

Western blot analysis was performed as previously described with a minor change (Wu et al., 2021). In short, 48 h after inhibition using *Ints7* MO oligos, BM-MSC protein lysates were separated by 10% sodium dodecyl sulfate (SDS)-polyacrylamide gel electrophoresis (SDS-PAGE), transferred to 0.22-μm polyvinylidene difluoride membranes (Millipore, Billerica, MA, United States) and incubated with specific anti-INTS7 (Proteintech, Chicago, IL, United States) or anti-ABCD3 (Abcam, Cambridge, MA, United States) antibodies. A beta-tubulin antibody (Beyotime, Nantong, China) was used as an internal control. After incubation with horseradish peroxidase (HRP)-conjugated secondary antibodies (Thermo Scientific, Waltham, MA, United States), signals were detected by enhanced chemiluminescence substrate (Thermo Scientific). The resulting bands were analyzed using Image-Pro Plus Software.

### Immunofluorescence Assays

Bone marrow mesenchymal stem cells were fixed in 4% (w/v) paraformaldehyde (PFA), blocked with 1% bovine serum albumin (BSA, w/v), and then reacted at 4°C overnight with anti-INTS7 primary antibody (Proteintech), as described previously (Shen et al., 2019). After washing with phosphate-buffered saline (PBS), the samples were incubated with a secondary fluorescent antibody (Thermo Scientific) for 1 h at room temperature. Slides were mounted using VECTASHIELD mounting medium with 4′,6-diamidino-2-phenylindole (DAPI; VECTOR, Burlingame, CA, United States). Sections were analyzed under a confocal laser-scanning microscope (Zeiss LSM800, Carl Zeiss, Oberkochen, Germany). Finally, five randomly selected views in each well were imaged and calculated for quantification of fluorescence intensity.

### CCK-8 and Ethynyldeoxyuridine (Edu) Experiments

After 48 h of INTS7/ABCD3/HDLBP inhibition, BM-MSCs were inoculated into 96-well plates (3000 cells/well). The cell proliferation capacity was evaluated every 24 h using Cell counting kit-8 (Beyotime). Ethynyl-2-deoxyuridine (EdU) experiments were performed using an EdU labeling and detection kit (RiboBio, Guangzhou, China), according to the manufacturer’s instructions. BM-MSCs were treated with 50 μM EdU labeling medium and incubated for another 2 h. Next, cells were fixed with 4% paraformaldehyde and permeated using 0.5% Triton X-100. Anti-EdU working solution and DAPI staining solution were added. EdU-positive cells were observed and counted under a confocal laser-scanning microscope (Zeiss LSM800, Carl Zeiss, Oberkochen, Germany).

### Flow-Cytometric Analysis

Bone marrow mesenchymal stem cells were harvested for cell-cycle analysis and fixed with cold ethanol overnight at 4°C. The cell suspension was centrifuged, and the collected pellets were washed with PBS and resuspended in 500 μl PBS. Cells were stained with propidium iodide in the dark using the CycleTEST™ PLUS DNA Reagent Kit (BD Biosciences, Franklin Lakes, NJ, United States), following the manufacturer’s protocol, and analyzed as previously described (Gao et al., 2020). The percentages of cells in the G0/G1, S, and G2/M phases were counted.

### Terminal Deoxynucleotidyl Transferase-Mediated dUTP Nick End Labeling (Tunel) Assays

Apoptotic cells were detected by a terminal deoxynucleotidyl transferase-mediated dUTP nick-end labeling (TUNEL; BrightRed Apoptosis Detection Kit, Vazyme), as previously described (Zhao et al., 2019). Briefly, BM-MSCs were fixed with 4% (w/v) PFA and treated with proteinase K (10 μg/ml) for 10 min at room temperature, followed by incubation with BrightRed Labeling Buffer for 45 min at 37°C. Cells were washed with PBS three times then stained with DAPI. Images were captured, and TUNEL-positive cells were counted under a confocal laser-scanning microscope (Zeiss LSM800, Carl Zeiss, Oberkochen, Germany).

### Osteogenesis and Adipogenesis Induced-Differentiation of Bone Marrow Mesenchymal Stem Cells

An osteogenesis-inducing differentiation medium kit (No: MUBMX-90021) was purchased from Cyagen Biosciences to enhance osteoblastic differentiation. Differentiated osteoblasts were stained with Alizarin Red S dye solution. An adipogenesis-inducing differentiation medium kit (No: MUBMX-90031) was purchased from Cyagen Biosciences to enhance adipogenic differentiation. Differentiated adipocytes were stained with Oil Red O dye solution.

### Immunoprecipitation (IP) Assays

Briefly, 5 μg of anti-INTS7 antibody (Proteintech) was incubated with total BM-MSC lysates for 2 h, followed by the addition of 20 μl of washed protein A/G beads (Santa Cruz Biotechnology) and incubated for another 16 h at 4°C. Beads were then pelleted and washed three times in 20× bed volume of lysis buffer. The bound protein was eluted by heating the beads at 95°C for 5 min with 2× SDS buffer. Finally, anti-INTS7 (Proteintech), anti-ABCD3 (Abcam), or anti-HDLBP (Proteintech) antibodies were used to detect the presence of each protein in the immunoprecipitation (IP) product. Inputs represented approximately 1/10 of the extract volume used for the IP experiment.

### Liquid Chromatography-Mass Spectrometry Analysis

The gel particles were cut into blocks approximately 1 mm^3^ in size for in-gel digestion. Gel particles were first washed with deionized water, 50% acetonitrile (can), and 100% ACN. Next, the proteins were reduced and alkylated for 45 min at room temperature in the dark. The gel particles were then thoroughly washed, dried naturally, rehydrated at 4°C for 30 min, and subjected to protein digestion at 37°C for 12 h. Trifluoroacetic acid (TFA), at a final concentration of 0.1%, was added to stop the digestion, and the supernatants were transferred to fresh tubes. All extracts and supernatants were combined and dried in a SpeedVac. The digested peptides were desalted with StageTip (Thermo Scientific) and analyzed using an LTQ Orbitrap Velos mass spectrometer (Thermo Scientific). We searched the tandem mass spectrometry (MS/MS) spectra using the UniProt protein database to identify proteins, with a false discovery rate of 1% for both peptides and proteins. Three replicates were performed. For label-free quantification, the protein expression levels were estimated using the iBAQ algorithm embedded in MaxQuant. The annotated proteins are listed in Supplementary Table 2. The data presented in the study are deposited in the ProteomeXchange Consortium via PRIDE partner repository, accession number PXD028817. The data can be accessed using the following details: Username: reviewer_pxd028817@ebi.ac.uk; Password: fyaTWuvX

### Transactional Analysis of INTS7 and ABCD3/HDLBP

InterPro tool (Blum et al., 2021) was used to intercept key or representative domain sequences during prediction, and 3D models were constructed accordingly. Robetta (Yang et al., 2020), a 3D protein structure prediction software, was used to model the 3D structure of the ligand (ABCD3: ABC transporter-like domain/HDLBP: KH domain) and receptor (INTS7: Armadillo-type fold superfamily) sequences. We used the HawkDock protein complex prediction tool (Weng et al., 2019) to analyze the interaction between the ligand and receptor and optimize the interface between the interacting residues using the built-in molecular mechanics with generalized Born and surface area solvation (MM/GBSA) algorithm. PyMOL (version: 2.1.0) software was used to visualize the results and annotate the interaction residues.

### Cellular Reactive Oxygen Species Analysis

After INTS7/ABCD3 inhibition for 48 h, BM-MSCs were incubated with 25 μM 2′,7′–dichlorofluorescein diacetate (DCFDA, Invitrogen, Carlsbad, CA, United States) for 45 min at 37°C. Cells were then washed with PBS, and the fluorescence intensities were immediately measured on a fluorescent plate reader or a confocal laser-scanning microscope (Zeiss LSM800, Carl Zeiss), as described previously (Yu et al., 2021).

### Statistical Analysis

Experiments were independently repeated at least three times. Data are presented as the mean ± standard deviation (SD). Student’s *t*-test and one-way analysis of variance (ANOVA) were used to evaluate significant differences between groups, *p < 0.05; ^**^p < 0.01; ^***^p < 0.001.

## Results

### INTS7 Inhibition Affects Bone Marrow Mesenchymal Stem Cell Proliferation and Apoptosis *in vitro*

**FIGURE 1:**
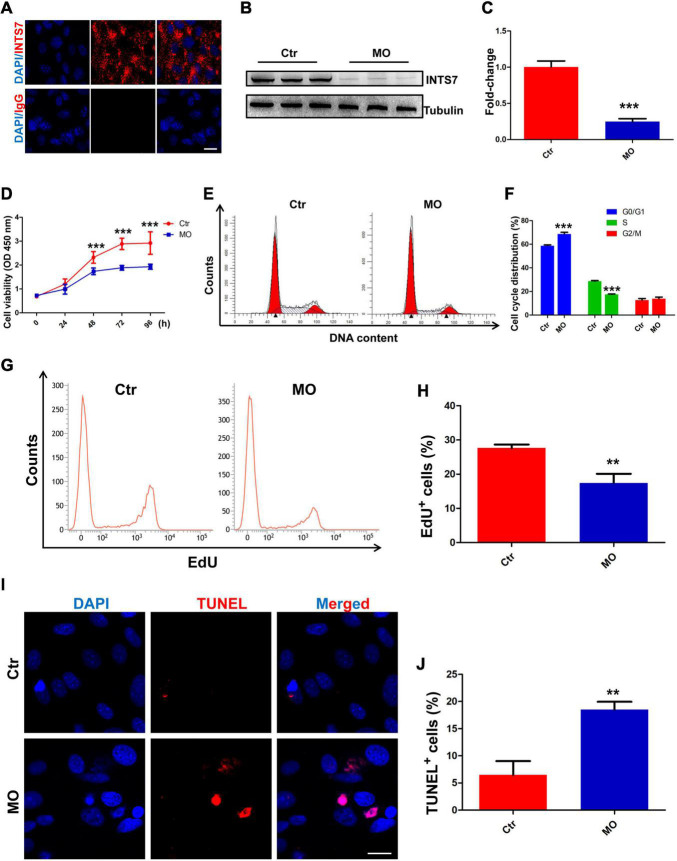
INTS7 inhibition affects BM-MSC proliferation and apoptosis *in vitro*. **(A)** Immunofluorescent staining was performed to detect the subcellular localization of INTS7 protein. INTS7 protein is labeled in red, and the cell nuclei are labeled in blue. **(B,C)** Western blot analysis of INTS7 protein after treatment with *Inst7*-MO oligos or negative control MO oligos (Ctr) for 48 h, *n* = 3. **(D)** Cell counting kit-8 (CCK-8) assays were used to determine the proliferation capacity of INTS7-depleted BM-MSCs, *n* = 6. **(E,F)** Flow cytometric analysis was used to determine the cell-cycle distribution of BM-MSCs after the treatment with *Ints7*-MO oligos for 48 h, *n* = 3. **(G,H)** An EdU-based flow cytometric analysis was conducted to evaluate EdU-positive BM-MSCs in the INTS7-depleted group compared with the Ctr group after treatment for 48 h, *n* = 3. **(I,J)** TUNEL assays were performed to determine BM-MSC apoptosis after INTS7 suppression for 48 h. The TUNEL-positive cells are labeled in red, and the cell nuclei are labeled in blue, *n* = 3. Scale bar, 20 μm. **p < 0.01; ***p < 0.001, Student’s *t*-test.

To explore the biological behaviors and potential molecular mechanisms through which INTS7 exerts effects in BM-MSCs derived from C57BL/6 mice, immunofluorescent staining was performed to characterize the subcellular localization of INTS7. We found that INTS7 protein was primarily localized in the cytoplasm rather than the nucleus, indicating that INTS7 may exert functions at the post-transcriptional or translational level (Figure 1A). Next, INTS7 was inhibited using *Inst7*-MO oligos, which reduced INST7 protein levels to 25% that of the negative control MO (Ctr; Figures 1B,C). CCK-8 assays showed that BM-MSC viability was prominently impaired in the *Inst7-*MO group compared with the Ctr group (Figure 1D). Flow cytometric analysis suggested that INST7 depletion resulted in cell-cycle arrest at the G0/G1 phase (Figures 1E,F). To further understand whether cell proliferation or apoptosis regulation was involved in observed effects on BM-MSC growth following INST7 depletion, an EdU-based flow cytometry analysis was performed, which revealed that the proportion of EdU-positive BM-MSCs in the INTS7-depleted group was significantly reduced compared with the Ctr group (Figures 1G,H). In addition, TUNEL staining analysis further indicated that INTS7 suppression could induce BM-MSC apoptosis (Figures 1I,J). These data suggested that INTS7 significantly promoted C57BL/6 mouse BM-MSC proliferation *in vitro*, partially due to an accelerated cell-cycle course and the attenuation of apoptotic behavior.

### The Effects of INTS7 on Osteoblast and Adipocyte Differentiation Among Bone Marrow Mesenchymal Stem Cells

**FIGURE 2:**
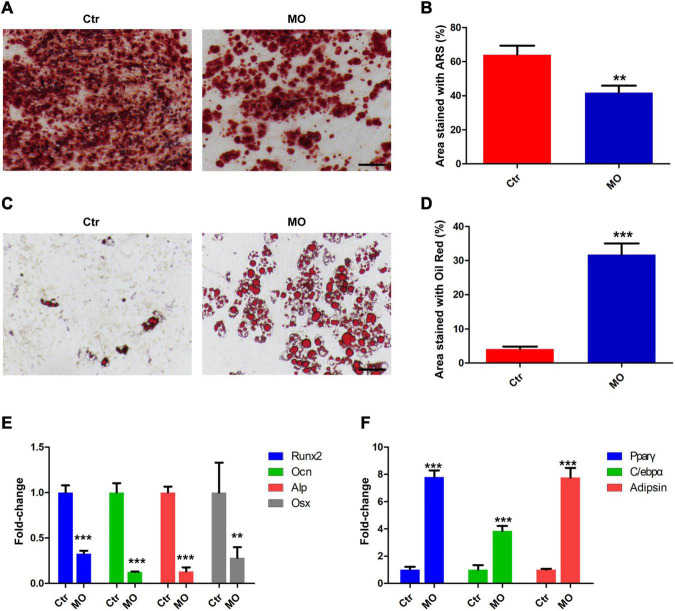
The effects of INTS7 on osteoblast and adipocyte differentiation in BM-MSCs. **(A)** Alizarin Red S dye solution was used to stain differentiated osteoblasts. **(B)** Quantification of the Alizarin Red S-stained areas in **(A)**, *n* = 3. **(C)** Oil Red O dye solution was used to stain differentiated adipocytes. **(D)** Quantification of the Oil Red O–stained areas in **(C)**, *n* = 3. **(E)** The mRNA levels of facilitated factors responsible for osteogenic differentiation were detected by QRT-PCR after INTS7 inhibition, *n* = 3. **(F)** The mRNA levels of effective molecules regulating adipocyte differentiation were detected by QRT-PCR after INTS7 inhibition, *n* = 3. Scale bar, 100 μm. **p < 0.01; ***p < 0.001, Student’s *t*-test.

To evaluate the effects of INTS7 on osteoblast and adipocyte differentiation among BM-MSCs, Alizarin Red S and Oil Red O dye solutions were, respectively, used to identify osteoblasts and adipocytes. As shown in Figure 2, the ability of BM-MSCs to differentiate into osteoblasts was significantly weakened by INTS7 inhibition (Figures 2A,B), whereas the number of differentiated adipocytes derived from BM-MSCs presented a five-fold increase (Figures 2C,D). Subsequently, the expression levels of several key transcription factors and signaling molecules involved in either osteoblast or adipocyte differentiation in BM-MSCs were detected in the Ctr and *Inst7*-MO groups, which revealed the downregulation of several facilitating factors involved in osteogenic differentiation following INST7 suppression, including *Runx2*, Osteocalcin (*Ocn*), alkaline phosphatase (*Alp*), and Osterix (*Osx*) (Figure 2E). By contrast, the effective molecules (PPARγ, C/EBPα, and adipsin) regulating adipocyte differentiation were significantly increased in the *Inst7*-MO group compared with the Ctr group (Figure 2F). Collectively, these results implied that INTS7 inhibition in BM-MSCs could decrease osteoblastic differentiation and accelerate adipocytic differentiation.

### INTS7 Interacts With ABCD3 and High-Density Lipoprotein-Binding Protein in Bone Marrow Mesenchymal Stem Cells

**FIGURE 3:**
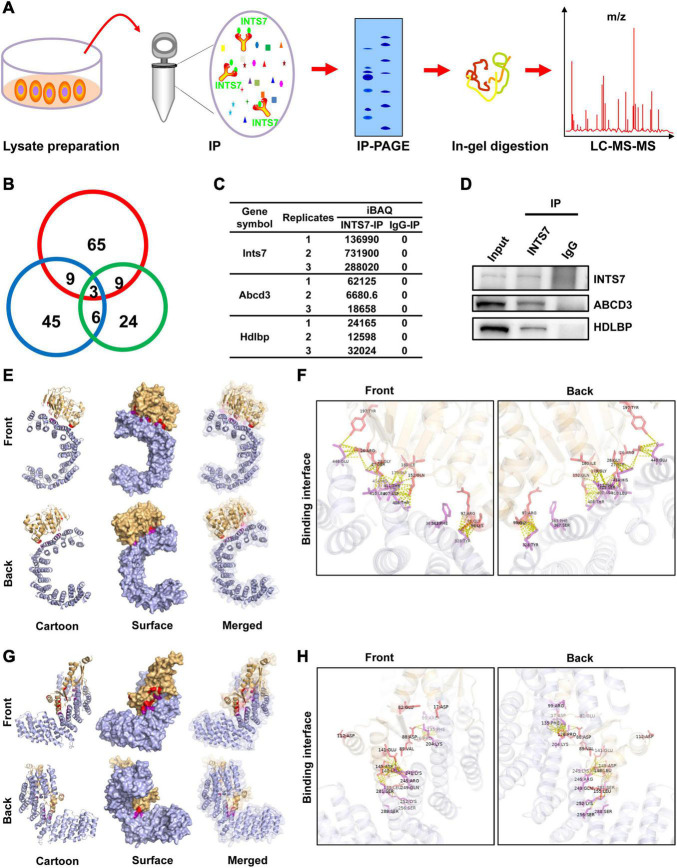
INTS7 interacts with ABCD3 and HDLBP in BM-MSCs. **(A)** The procedures used to identify INTS7-interacting proteins in BM-MSCs: total endogenous proteins were collected, subjected to INTS7 antibody-based immunoprecipitation, IP-PAGE, in-gel digestion, liquid chromatography separation, and mass spectrometry analysis. **(B)** Three replicates in **(A)** were performed, and the quantities of co-occurring proteins were evaluated. **(C)** The protein expression levels were estimated by the iBAQ algorithm embedded in MaxQuant. **(D)** Protein extracts were incubated with an anti-INTS7 antibody for IP assays, followed by western blot analysis with anti- INTS7, anti-ABCD3, or anti-HDLBP antibodies. **(E)** Visualization of the docking interface between INST7 and ABCD3. The front and back views of the docked protein complex between ABCD3 (orange) and INTS7 (blue). The cartoon mode represents the backbone and the secondary structures of the corresponding proteins. The surface mode displays the solvent-accessible surface area. The merged mode combines the cartoon and the transparent surface views. **(F)** The interface view shows the binding residues (in stick mode) at the interface between ABCD3 and INTS7. The interacting residues are colored in red and purple for ABCD3 and INTS7, respectively. The molecular contacts between interacting residues are indicated by dashed yellow lines. **(G)** Visualization of the docking interface between INTS7 and HDLBP. The front and back views of the docked protein complex between HDLBP (orange) and INTS7 (blue). **(H)** The interface view shows the binding residues (in stick mode) at the interface between HDLBP and INTS7. The interacting residues are colored in red and purple for HDLBP and INTS7, respectively. The molecular contacts between the interacting residues are demonstrated by dashed yellow lines.

To identify proteins that interact with INTS7 in BM-MSCs, total endogenous proteins were collected, subjected to IP experiments using an anti-INTS7 antibody, and subjected to IP-PAGE, in-gel digestion, liquid chromatography separation, and mass spectrometry analysis (Figure 3A). Overall, three proteins, INTS7, ABCD3, and HDLBP, were successfully identified and quantified in three independently repeated experiments (Figures 3B,C). Co-IP assays confirmed the interaction between INTS7 and ABCD3/HDLBP proteins (Figure 3D), suggesting that INTS7 affects the proliferation and differentiation of BM-MSCs potentially through a cooperative mechanism involving ABCD3 or HDLBP proteins. Moreover, 3D model construction and bioinformatics analysis were performed to characterize the interactions between INTS7 and ABCD3/HDLBP proteins. The predicted 3D structural model for the protein interaction docking between INTS7 and ABCD3 is exhibited in Figure 3E, and the binding interface from both front and back perspectives is further visualized in Figure 3F. Similarly, Figures 3G,H separately displayed the 3D protein interaction docking and visual binding interface, respectively, between INTS7 and HDLBP.

### The Effects of ABCD3/HDLBP on Bone Marrow Mesenchymal Stem Cell Proliferation and Apoptosis *in vitro*

**FIGURE 4:**
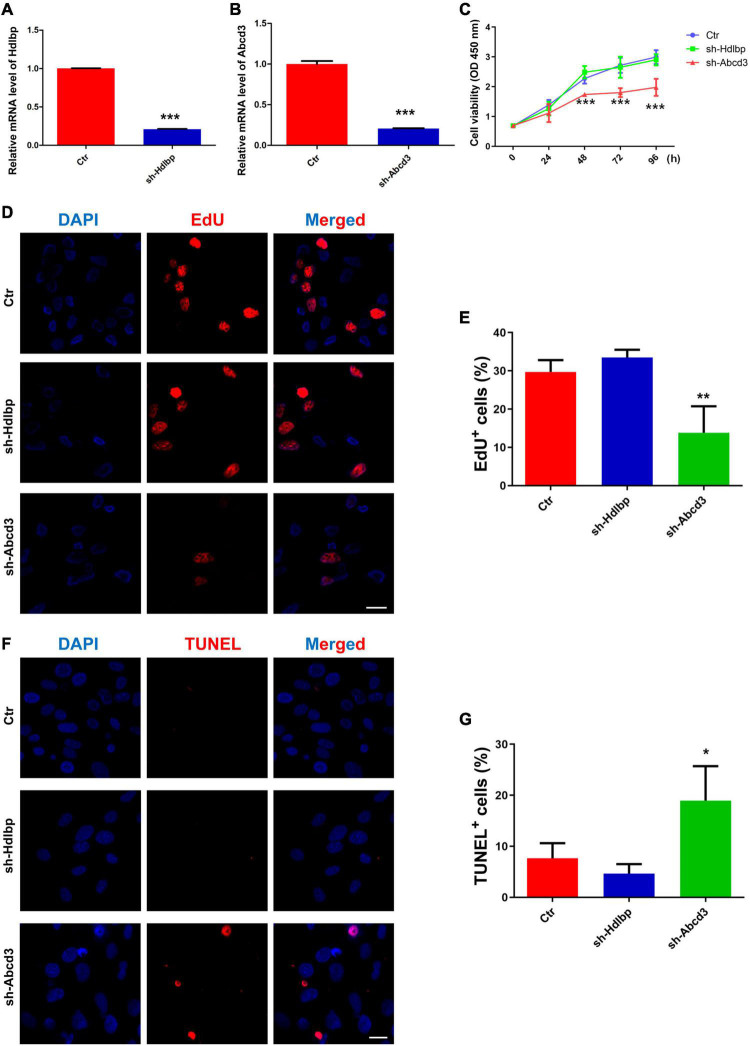
The effects of HDLBP/ABCD3 on BM-MSC proliferation and apoptosis *in vitro*. **(A,B)** Relative mRNA levels of *Hdlbp* and *Abcd3* were significantly reduced by each respective shRNA for 48 h, *n* = 3. **(C)** CCK-8 assays were used to determine the proliferative capacity of HDLBP- and ABCD3-depleted BM-MSCs, *n* = 6. **(D,E)** EdU staining was conducted to evaluate EdU-positive BM-MSCs in both HDLBP- and ABCD3-depleted groups compared with the negative control after treatment for 48 h. The EdU-positive cells are labeled in red, and cell nuclei are labeled in blue, *n* = 3. **(F,G)** TUNEL assays were performed to determine BM-MSC apoptosis after HDLBP or ABCD3 suppression for 48 h. The TUNEL-positive cells are labeled in red, and cell nuclei are labeled in blue, *n* = 3. Scale bar, 20 μm. *p < 0.05; **p < 0.01; ***p < 0.001. For **(A,B)**, Student’s *t*-test; for **(C,E,G)**, one-way ANOVA.

The biological functions of ABCD3/HDLBP were next examined in BM-MSCs. First, *Hdlbp* and *Abcd3* were significantly knocked down using targeted shRNAs (Figures 4A,B). CCK8 assays indicated that *Abcd3* silencing could prominently impair BM-MSC viability, whereas *Hdlbp* inhibition had no impact on BM-MSC growth (Figure 4C). Next, EdU staining revealed that the proportion of EdU-positive BM-MSCs in the sh-*Abcd3* group was significantly decreased compared with that in the negative control (Ctr) group. However, the number of EdU-positive cells was not altered after *Hdlbp* downregulation (Figures 4D,E). Analogously, TUNEL staining analysis demonstrated that apoptosis was induced in BM-MSCs in the sh-*Abcd3* group but not in the sh-*Hdlbp* group compared with the Ctr group (Figures 4F,G). These data supported an active role for ABCD3 in the regulation of BM-MSC growth.

### The Effects of ABCD3/HDLBP on Osteoblast and Adipocyte Differentiation of Bone Marrow Mesenchymal Stem Cells

**FIGURE 5:**
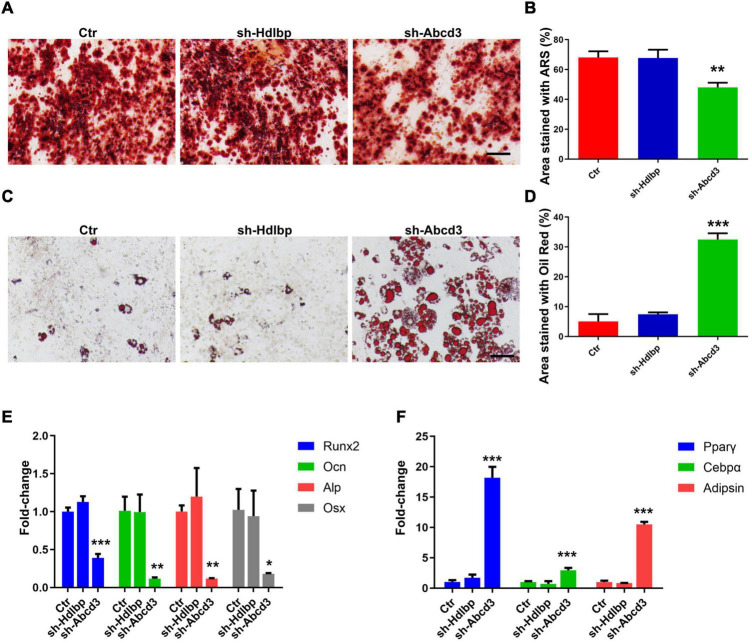
The effects of HDLBP and ABCD3 on osteoblast and adipocyte differentiation in BM-MSCs. **(A)** Alizarin Red S dye solution was used to stain differentiated osteoblasts after HDLBP or ABCD3 suppression. **(B)** Quantification of the Alizarin Red S-stained areas in **(A)**, *n* = 3. **(C)** Oil Red O dye solution was used to stain differentiated adipocytes after HDLBP or ABCD3 inhibition. **(D)** Quantification of the Oil Red O-stained areas in **(C)**, *n* = 3. **(E)** The mRNA levels of facilitating factors involved in osteogenic differentiation were detected by QRT-PCR after HDLBP or ABCD3 inhibition, *n* = 3. **(F)** The mRNA levels of effective molecules regulating adipocyte differentiation were detected by QRT-PCR after HDLBP or ABCD3 inhibition, *n* = 3. Scale bar, 100 μm, *p < 0.05; **p < 0.01; ***p < 0.001, one-way ANOVA.

Alizarin Red S and Oil Red O staining were performed to evaluate the effects of ABCD3/HDLBP on osteoblast and adipocyte differentiation in BM-MSCs. The results showed that the proportion of differentiated osteoblasts decreased by 30%, whereas a five-fold increase in the number of differentiated adipocytes was observed following *Abcd3* suppression (Figures 5A–D). As expected, *Hdlbp* suppression exerted no impacts on either osteoblast or adipocyte differentiation in BM-MSCs (Figures 5A–D). Molecule marker detection after *Abcd3* knockdown revealed the downregulation of factors (*Runx2*, *Ocn*, *Alp*, and *Osx*) associated with osteoblast differentiation and the increased expression of factors (PPARγ, C/EBPα, and adipsin) associated with adipocyte differentiation (Figures 5E,F). However, no changes in the expression levels of any of the examined markers were observed following *Hdlbp* depletion in BM-MSCs (Figures 5E,F). These data supported the finding that *Abcd3* inhibition activated BM-MSC differentiation into adipocytes and inhibited osteogenic differentiation, whereas *Hdlbp* had no involvement in BM-MSC differentiation.

### The Suppression of Oxidative Stress Is Potentially Involved in the INTS7–ABCD3 Co-regulatory Effects on Bone Marrow Mesenchymal Stem Cell Proliferation and Differentiation

**FIGURE 6:**
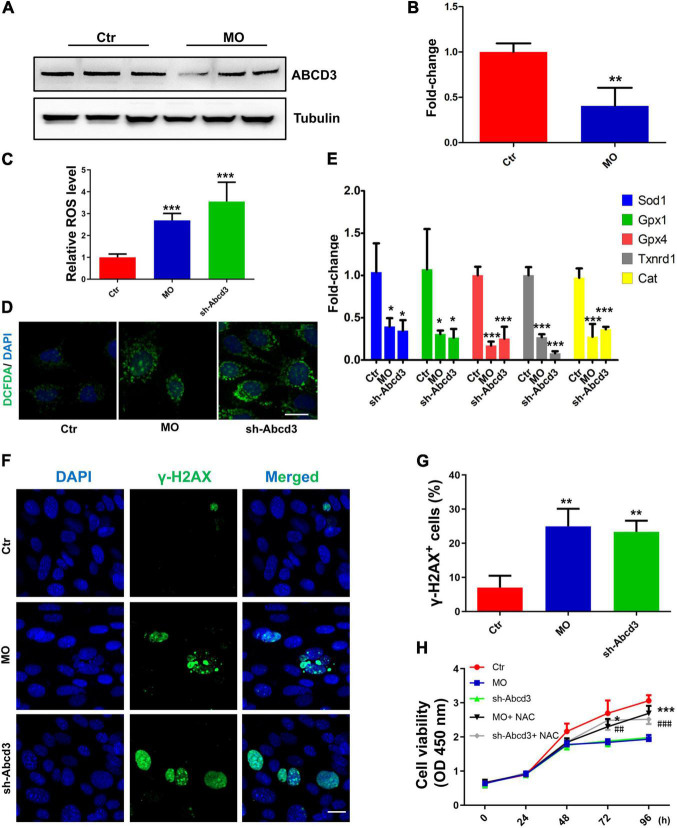
Low levels of oxidative stress are potentially responsible for the INTS7–ABCD3 co-regulatory effects on BM-MSC proliferation and differentiation. **(A)** Western blot analysis of ABCD3 protein after the treatment with *Ints7*-MO oligos or negative control MO for 48 h. Beta-tubulin protein was used as an internal control. **(B)** Quantification of ABCD3 protein levels in **(A)**, *n* = 3. **(C,D)** Levels of ROS in BM-MSCs treated with *Ints7*-MO oligos or *Abcd3*-shRNAs for 48 h, which were measured on a fluorescent plate reader **(C)** or a confocal laser-scanning microscope **(D)**, *n* = 6. **(E)** The mRNA levels of several endogenous antioxidants were detected by QRT-PCR after INTS7 or ABCD3 inhibition for 48 h, *n* = 3. **(F)** Immunostaining of γ-H2AX in BM-MSCs treated with *Ints7*-MO oligos or *Abcd3*-shRNAs for 48 h. The γ-H2AX–positive cells are labeled in green, and cell nuclei are labeled in blue. **(G)** Quantification of γ-H2AX–positive cells in **(F)**, *n* = 3. **(H)** CCK-8 assays were used to determine the proliferative capacity of BM-MSCs treated as indicated. N-acetylcysteine (NAC) was used at the concentration of 500 μM. *, compared with *Ints7*-MO; ^#^, compared with *Abcd3*-shRNAs, *n* = 6. Scale bar, 20 μm, *p < 0.05; **p < 0.01; ***p < 0.001, ^##^*p* < 0.01; ^###^*p* < 0.001. For **(B)**, Student’s *t*-test; for **(C,E,G,H)**, one-way ANOVA.

Western blot analysis found that the quantity of ABCD3 protein was significantly reduced in response to *Ints7* silencing (Figures 6A,B), which indicated that ABCD3 was a potential downstream target of INTS7, and the INTS7–ABCD3 pathway may exert co-regulatory effects in BM-MSCs. As previously reported, MSCs are highly sensitive to oxidative stress compared with differentiated cell types, and excessive ROS may impair the differentiation and proliferation capacity of MSCs (Ko et al., 2012; Denu and Hematti, 2016). To explore whether oxidative stress suppression was involved in the regulatory effects of INTS7–ABCD3 on BM-MSC proliferation and osteoblastic differentiation, we examined ROS and antioxidant quantities in INTS7- or ABCD3-depleted BM-MSCs. The results showed that the ROS levels in the treatment groups were significantly upregulated compared with those in the control group (Figures 6C,D). In addition, the expression levels of antioxidants (including *Sod1*, *Gpx1*, *Gpx4*, *Txnrd1*, and *Cat*) decreased significantly in response to both INTS7 and ABCD3 depletion (Figure 6E). DNA damage is associated with the upregulation of oxidative stress. We performed immunostaining to detect the histone γ-H2AX (a marker of DNA double-strand breaks) to confirm this phenomenon. Figures 6F,G show that the percentage of γ-H2AX–positive cells increased following the depletion of both INTS7 and ABCD3. Moreover, adding of the antioxidant scavenger N-acetylcysteine (NAC) after INTS7/ABCD3 knockdown could both partially rescue the cell viability of BM-MSCs (Figure 6H). These findings suggested that the low levels of oxidative stress are potentially responsible for the INTS7–ABCD3 co-regulatory effects observed for BM-MSC proliferation and differentiation.

## Discussion

Bone marrow mesenchymal stem cells are pluripotent cells located in the bone marrow and are capable of differentiating into osteoblasts, adipocytes, or chondrocytes (Hu et al., 2018). BM-MSCs have been shown to repair bone damage and enhance bone regeneration, and the role played by osteoblastic differentiation appears to be particularly influential in the repair of constant microfractures and the maintenance of the dynamic properties of bone (Bielby et al., 2007). Multiple endogenous bioactive molecules secreted by BM-MSCs have been demonstrated to form a regulatory network that generates an optimal regenerative microenvironment for either osteoblast or adipocyte differentiation among BM-MSCs (Caplan, 2007).

Here, we focused on a new stimulatory factor, INTS7, in C57BL/6 mouse BM-MSCs, which was associated with cell growth and differentiation. Previous studies have reported that the transcription of the human *INTS7* gene was significantly altered in several cancers, and *INTS7* depletion was shown to induce cell-cycle arrest (Federico et al., 2017). Moreover, Cecilia Cotta-Ramusino et al. (2011), showed that human INTS7 could interact with single-stranded DNA-binding protein 1 (SSB1), forming a complex that was recruited to DNA damage sites and participated in the DNA damage response. However, in our study, INTS7 protein was primarily localized to the cytoplasm rather than the nucleus in mouse BM-MSCs and appeared to stimulate the proliferation and osteoblastic differentiation of C57BL/6 mouse BM-MSCs by suppressing oxidative stress.

In this study, the INTS7-IP assays from BM-MSC lysates and the ensuing mass spectrometry analysis of IP products revealed the interaction between INTS7 and ABCD3, which both exerted regulatory effects on BM-MSC proliferation and differentiation. ABCD3, also known as 70-kDa peroxisomal membrane protein (PMP70), participates in the peroxisomal import of fatty acids or fatty acyl-CoAs (Imanaka et al., 2000; Kawaguchi and Morita, 2016; Yu et al., 2018). Peroxisomes are primarily involved in ROS regulation and lipid metabolism, processes that are regulated by proteins belonging to the peroxin (PEX) family (Colasante et al., 2017). Across known PEX proteins, PEX2 overexpression has been shown to induce pexophagy, and ABCD3 protein has been demonstrated to be a downstream target of PEX2 (Sargent et al., 2016). The literature suggests that the accumulation of ROS in peroxisomes can trigger pexophagy (Lee et al., 2018). These data suggest that the ROS–PEX2–ABCD3 axis could serve as an effective regulatory axis for pexophagy. Our research further confirmed the involvement of ROS suppression in the ABCD3 regulatory effects on BM-MSC proliferation and differentiation, consistent with the observed regulatory mechanism of INTS7 in BM-MSCs.

Previous research has demonstrated the significance of oxidative stress for MSC longevity and function. In general, a low basal level of ROS is both necessary and advantageous for MSC proliferation, differentiation, and survival (Kobayashi and Suda, 2012; Atashi et al., 2015). However, compared with differentiated cell types, undifferentiated MSCs exhibited reduced antioxidant capacity and were more sensitive to oxidative stress. Excessive ROS could impair MSC self-renewal, proliferation, and osteogenic differentiation while simultaneously increasing senescence and adipogenic differentiation (Ko et al., 2012; Choo et al., 2014); concurrently, endogenous antioxidants may stimulate MSC proliferation (Zou et al., 2004). Studies have reported that the addition of exogenous H_2_O_2_ reduced osteogenic differentiation in human and murine MSCs (Mody et al., 2001; Chen et al., 2008). Kanda et al. (2011) indicated that the ROS scavenger N-acetylcysteine (NAC) inhibited adipogenesis in the mouse MSC cell line 10T1/2. Taken together, these findings suggest that excessive ROS can suppress MSC osteogenesis and stimulate adipogenesis. In this study, we further identified that INTS7 and ABCD3 downregulation could both increase ROS quantities and decrease antioxidant levels, indicating the potential involvement of oxidative stress in the INTS7–ABCD3 co-regulatory effects on BM-MSC proliferation and differentiation. However, the limitation of this study was that we have not offered evidence demonstrating the stimulatory effects of INTS7 and ABCD3 in human BM-MSC growth and osteogenic differentiation, as well as did not validate the result in animal model, which require further verification in future studies.

## Conclusion

Here, we focused on a novel factor, INTS7, and its cooperative protein ABCD3, which were able to induce increased osteoblast differentiation and decreased adipocyte differentiation in C57BL/6 mouse BM-MSCs through the suppression of oxidative stress. These findings verified the essential role played by oxidative stress in the INTS7–ABCD3 co-regulatory effects on BM-MSC biological behaviors and provides new potential candidates for osteoporosis therapy. However, we have not offered evidence demonstrating the stimulatory effects of INTS7 and ABCD3 in human BM-MSC growth and osteogenic differentiation, which represents a limitation of the present study and requires further verification in future studies.

## Data Availability Statement

The data presented in the study are deposited in the ProteomeXchange Consortium via the PRIDE partner repository, accession number PXD028817.

## Author Contributions

YL, XY, and AH performed most of the experiments. XZ, YW, WG, RX, and SL performed some of the experiments. YL, HH, and BZ analyzed the data. GC and YX initiated the project and designed the experiments. YL and BZ wrote the manuscript. All authors read and approved the final manuscript.

## Conflict of Interest

The authors declare that the research was conducted in the absence of any commercial or financial relationships that could be construed as a potential conflict of interest.

## Publisher’s Note

All claims expressed in this article are solely those of the authors and do not necessarily represent those of their affiliated organizations, or those of the publisher, the editors and the reviewers. Any product that may be evaluated in this article, or claim that may be made by its manufacturer, is not guaranteed or endorsed by the publisher.

## References

[B1] Atashi F. Modarressi A. Pepper M. S. (2015). The role of reactive oxygen species in mesenchymal stem cell adipogenic and osteogenic differentiation: a review. *Stem Cells Dev.* 24 1150–1163. 10.1089/scd.2014.0484 25603196 PMC4424969

[B2] Augello A. De Bari C. (2010). The regulation of differentiation in mesenchymal stem cells. *Hum. Gene Ther.* 21 1226–1238. 10.1089/hum.2010.173 20804388

[B3] Bielby R. Jones E. McGonagle D. (2007). The role of mesenchymal stem cells in maintenance and repair of bone. *Injury* 38(Suppl. 1), S26–S32. 10.1016/j.injury.2007.02.007 17383482

[B4] Blum M. Chang H. Y. Chuguransky S. Grego T. Kandasaamy S. Mitchell A. (2021). The InterPro protein families and domains database: 20 years on. *Nucleic Acids Res.* 49 D344–D354. 10.1093/nar/gkaa977 33156333 PMC7778928

[B5] Burge R. Dawson-Hughes B. Solomon D. H. Wong J. B. King A. Tosteson A. (2007). Incidence and economic burden of osteoporosis-related fractures in the United States, 2005-2025. *J. Bone Miner. Res.* 22 465–475. 10.1359/jbmr.061113 17144789

[B6] Caplan A. I. (2007). Adult mesenchymal stem cells for tissue engineering versus regenerative medicine. *J. Cell. Physiol.* 213 341–347. 10.1002/jcp.21200 17620285

[B7] Chen C. T. Shih Y. R. Kuo T. K. Lee O. K. Wei Y. H. (2008). Coordinated changes of mitochondrial biogenesis and antioxidant enzymes during osteogenic differentiation of human mesenchymal stem cells. *Stem Cells.* 26 960–968. 10.1634/stemcells.2007-0509 18218821

[B8] Chen Q. H. Wu F. Liu L. Chen H. B. Zheng R. Q. Wang H. L. (2020). Mesenchymal stem cells regulate the Th17/Treg cell balance partly through hepatocyte growth factor *in vitro*. *Stem Cell Res. Ther.* 11:91. 10.1186/s13287-020-01612-y 32111238 PMC7049226

[B9] Choo K. B. Tai L. Hymavathee K. S. Wong C. Y. Nguyen P. N. (2014). Oxidative stress-induced premature senescence in Wharton’s jelly-derived mesenchymal stem cells. *Int. J. Med. Sci.* 11 1201–1207. 10.7150/ijms.8356 25249788 PMC4166865

[B10] Colasante C. Chen J. Ahlemeyer B. Bonilla-Martinez R. Karnati S. Baumgart-Vogt E. (2017). New insights into the distribution, protein abundance and subcellular localisation of the endogenous peroxisomal biogenesis proteins PEX3 and PEX19 in different organs and cell types of the adult mouse. *PLoS One* 12:e183150. 10.1371/journal.pone.0183150 28817674 PMC5560687

[B11] Cotta-Ramusino C. McDonald E. R. Hurov K. Sowa M. E. Harper J. W. Elledge S. J. (2011). A DNA damage response screen identifies RHINO, a 9-1-1 and TopBP1 interacting protein required for ATR signaling. *Science* 332 1313–1317. 10.1126/science.1203430 21659603 PMC4357496

[B12] Denu R. A. Hematti P. (2016). Effects of oxidative stress on mesenchymal stem cell biology. *Oxid. Med. Cell. Longev.* 2016:2989076. 10.1155/2016/2989076 27413419 PMC4928004

[B13] Federico A. Rienzo M. Abbondanza C. Costa V. Ciccodicola A. Casamassimi A. (2017). Pan-Cancer mutational and transcriptional analysis of the integrator complex. *Int. J. Mol. Sci.* 18:936. 10.3390/ijms18050936 28468258 PMC5454849

[B14] Gao T. Lin M. Shao B. Zhou Q. Wang Y. Chen X. (2020). BMI1 promotes steroidogenesis through maintaining redox homeostasis in mouse MLTC-1 and primary Leydig cells. *Cell Cycle* 19 1884–1898. 10.1080/15384101.2020.1779471 32594840 PMC7469621

[B15] Hu L. Yin C. Zhao F. Ali A. Ma J. Qian A. (2018). Mesenchymal stem cells: cell fate decision to osteoblast or adipocyte and application in osteoporosis treatment. *Int. J. Mol. Sci.* 19:360. 10.3390/ijms19020360 29370110 PMC5855582

[B16] Imanaka T. Aihara K. Suzuki Y. Yokota S. Osumi T. (2000). The 70-kDa peroxisomal membrane protein (PMP70), an ATP-binding cassette transporter. *Cell Biochem. Biophys.* 32 Spring 131–138. 11330039 10.1385/cbb:32:1-3:131

[B17] Jiang Y. Zhang P. Zhang X. Lv L. Zhou Y. (2021). Advances in mesenchymal stem cell transplantation for the treatment of osteoporosis. *Cell Prolif.* 54:e12956. 10.1111/cpr.12956 33210341 PMC7791182

[B18] Johnell O. Kanis J. A. (2006). An estimate of the worldwide prevalence and disability associated with osteoporotic fractures. *Osteoporos. Int.* 17 1726–1733. 10.1007/s00198-006-0172-4 16983459

[B19] Kanda Y. Hinata T. Kang S. W. Watanabe Y. (2011). Reactive oxygen species mediate adipocyte differentiation in mesenchymal stem cells. *Life Sci.* 89 250–258. 10.1016/j.lfs.2011.06.007 21722651

[B20] Kawaguchi K. Morita M. (2016). ABC transporter subfamily d: distinct differences in behavior between ABCD1-3 and ABCD4 in subcellular localization, function, and human disease. *Biomed. Res. Int.* 2016:6786245. 10.1155/2016/6786245 27766264 PMC5059523

[B21] Ko E. Lee K. Y. Hwang D. S. (2012). Human umbilical cord blood-derived mesenchymal stem cells undergo cellular senescence in response to oxidative stress. *Stem Cells Dev.* 21 1877–1886. 10.1089/scd.2011.0284 22066510 PMC3396141

[B22] Kobayashi C. I. Suda T. (2012). Regulation of reactive oxygen species in stem cells and cancer stem cells. *J. Cell. Physiol.* 227 421–430. 10.1002/jcp.22764 21448925

[B23] Kobayashi H. Gao Y. Ueta C. Yamaguchi A. Komori T. (2000). Multilineage differentiation of Cbfa1-deficient calvarial cells *in vitro*. *Biochem. Biophys. Res. Commun.* 273 630–636. 10.1006/bbrc.2000.2981 10873656

[B24] Lane M. D. Lin F. T. MacDougald O. A. Vasseur-Cognet M. (1996). Control of adipocyte differentiation by CCAAT/enhancer binding protein alpha (C/EBP alpha). *Int. J. Obes. Relat. Metab. Disord.* 20(Suppl. 3), S91–S96. 8680485

[B25] Lee J. N. Dutta R. K. Maharjan Y. Liu Z. Q. Lim J. Y. Kim S. J. (2018). Catalase inhibition induces pexophagy through ROS accumulation. *Biochem. Biophys. Res. Commun.* 501 696–702. 10.1016/j.bbrc.2018.05.050 29753736

[B26] Li C. J. Cheng P. Liang M. K. Chen Y. S. Lu Q. Wang J. Y. (2015). MicroRNA-188 regulates age-related switch between osteoblast and adipocyte differentiation. *J. Clin. Invest.* 125 1509–1522. 10.1172/JCI77716 25751060 PMC4396470

[B27] Liu A. R. Liu L. Chen S. Yang Y. Zhao H. J. Liu L. (2013). Activation of canonical wnt pathway promotes differentiation of mouse bone marrow-derived MSCs into type II alveolar epithelial cells, confers resistance to oxidative stress, and promotes their migration to injured lung tissue *in vitro*. *J. Cell. Physiol.* 228 1270–1283. 10.1002/jcp.24282 23154940

[B28] Mody N. Parhami F. Sarafian T. A. Demer L. L. (2001). Oxidative stress modulates osteoblastic differentiation of vascular and bone cells. *Free Radic. Biol. Med.* 31 509–519. 10.1016/s0891-5849(01)00610-4 11498284

[B29] Moerman E. J. Teng K. Lipschitz D. A. Lecka-Czernik B. (2004). Aging activates adipogenic and suppresses osteogenic programs in mesenchymal marrow stroma/stem cells: the role of PPAR-gamma2 transcription factor and TGF-beta/BMP signaling pathways. *Aging Cell* 3 379–389. 10.1111/j.1474-9728.2004.00127.x 15569355 PMC1850101

[B30] Mulder J. E. Kolatkar N. S. LeBoff M. S. (2006). Drug insight: existing and emerging therapies for osteoporosis. *Nat. Clin. Pract. Endocrinol. Metab.* 2 670–680. 10.1038/ncpendmet0325 17143314

[B31] Nakashima K. Zhou X. Kunkel G. Zhang Z. Deng J. M. Behringer R. R. (2002). The novel zinc finger-containing transcription factor osterix is required for osteoblast differentiation and bone formation. *Cell* 108 17–29. 10.1016/s0092-8674(01)00622-5 11792318

[B32] Pietschmann P. Rauner M. Sipos W. Kerschan-Schindl K. (2009). Osteoporosis: an age-related and gender-specific disease–a mini-review. *Gerontology* 55 3–12. 10.1159/000166209 18948685

[B33] Sargent G. van Zutphen T. Shatseva T. Zhang L. Di Giovanni V. Bandsma R. (2016). PEX2 is the E3 ubiquitin ligase required for pexophagy during starvation. *J. Cell Biol.* 214 677–690. 10.1083/jcb.201511034 27597759 PMC5021090

[B34] Shen C. Yu J. Zhang X. Liu C. C. Guo Y. S. Zhu J. W. (2019). Strawberry Notch 1 (SBNO1) promotes proliferation of spermatogonial stem cells *via* the noncanonical Wnt pathway in mice. *Asian J. Androl.* 21 345–350. 10.4103/aja.aja_65_18 30198493 PMC6628735

[B35] Wang S. Qu X. Zhao R. C. (2012). Clinical applications of mesenchymal stem cells. *J. Hematol. Oncol.* 5:19. 10.1186/1756-8722-5-19 22546280 PMC3416655

[B36] Weng G. Wang E. Wang Z. Liu H. Zhu F. Li D. (2019). HawkDock: a web server to predict and analyze the protein-protein complex based on computational docking and MM/GBSA. *Nucleic Acids Res.* 47 W322–W330. 10.1093/nar/gkz397 31106357 PMC6602443

[B37] Wu Y. Wang T. Zhao Z. Liu S. Shen C. Li H. (2021). Retinoic acid induced protein 14 (Rai14) is dispensable for mouse spermatogenesis. *PeerJ* 9:e10847. 10.7717/peerj.10847 33643708 PMC7899019

[B38] Xu C. Wang J. Zhu T. Shen Y. Tang X. Fang L. (2016). Cross-Talking between PPAR and WNT signaling and its regulation in mesenchymal stem cell differentiation. *Curr. Stem Cell Res. Ther.* 11 247–254. 10.2174/1574888x10666150723145707 26201865

[B39] Yang D. Okamura H. Qiu L. (2018). Upregulated osterix expression elicited by Runx2 and Dlx5 is required for the accelerated osteoblast differentiation in PP2A Calpha-knockdown cells. *Cell Biol. Int.* 42 403–410. 10.1002/cbin.10902 29068100

[B40] Yang J. Anishchenko I. Park H. Peng Z. Ovchinnikov S. Baker D. (2020). Improved protein structure prediction using predicted interresidue orientations. *Proc. Natl. Acad. Sci. U S A.* 117 1496–1503. 10.1073/pnas.1914677117 31896580 PMC6983395

[B41] Yu J. Wu Y. Li H. Zhou H. Shen C. Gao T. (2021). BMI1 Drives Steroidogenesis through Epigenetically Repressing the p38 MAPK Pathway. *Front. Cell Dev. Biol.* 9:665089. 10.3389/fcell.2021.665089 33928089 PMC8076678

[B42] Yu T. Zhang H. Qi H. (2018). Transcriptome profiling analysis reveals biomarkers in colon cancer samples of various differentiation. *Oncol. Lett.* 16 48–54. 10.3892/ol.2018.8668 29928385 PMC6006489

[B43] Zhao D. Shen C. Gao T. Li H. Guo Y. Li F. (2019). Myotubularin related protein 7 is essential for the spermatogonial stem cell homeostasis *via* PI3K/AKT signaling. *Cell Cycle* 18 2800–2813. 10.1080/15384101.2019.1661174 31478454 PMC6773228

[B44] Zhuang H. Zhang X. Zhu C. Tang X. Yu F. Shang G. W. (2016). Molecular mechanisms of PPAR-gamma governing MSC osteogenic and adipogenic differentiation. *Curr. Stem Cell Res. Ther.* 11 255–264. 26027680 10.2174/1574888x10666150531173309

[B45] Zou X. Li H. Chen L. Baatrup A. Bunger C. Lind M. (2004). Stimulation of porcine bone marrow stromal cells by hyaluronan, dexamethasone and rhBMP-2. *Biomaterials* 25 5375–5385. 10.1016/j.biomaterials.2003.12.041 15130722

